# Relevance of a complete re-resection of contrast enhancing tumor in MGMT promotor non-methylated versus methylated recurrent glioblastomas

**DOI:** 10.1016/j.bas.2026.106047

**Published:** 2026-04-11

**Authors:** Dragan Jankovic, Santhosh G. Thavarajasingam, Ahmed Salih, Daniel Scurtu, Alice Dauth, Harald Krenzlin, Darius Kalasauskas, Andreas Kramer, Marc A. Brockmann, Clemens Sommer, Petra Leukel, Marcus Stockinger, Naureen Keric, Florian Ringel

**Affiliations:** a Department of Neurosurgery, University Medical Center Mainz, Mainz, Germany; b Department of Neurosurgery, LMU University Hospital, Munich, Germany; c Imperial Brain & Spine Initiative, Imperial College London, London, United Kingdom; d Faculty of Medicine, Imperial College London, London, United Kingdom; e Department of Neurosurgery, University Hospital Schleswig-Holstein, Luebeck, Germany; f Department of Neuroradiology, University Medical Center Mainz, Mainz, Germany; g Institute of Neuropathology, University Medical Center Mainz, Mainz, Germany; h Department of Radiooncology and Radiotherapy, University Medical Center Mainz, Mainz, Germany

**Keywords:** Recurrent glioblastoma, Extent of resection, MGMT promoter methylation, Re-Resection surgery, Survival outcome

## Abstract

**Introduction:**

Management of recurrent glioblastoma (rGB) requires an individualized approach, considering tumor location, functional status, and patient age. While lomustine chemotherapy remains the standard treatment for most patients, response is limited in MGMT non-methylated tumors.

**Research question:**

Does maximal surgical resection improve survival outcomes in recurrent glioblastoma patients with poor response to systemic therapy?

**Material and methods:**

We performed a retrospective analysis of rGB patients who underwent re-resection between January 2016 and December 2021. Clinical, radiological, and molecular data were collected. Extent of resection was defined by RANO criteria. Progression-free survival after re-resection (PFS2), reoperation survival (RS), and overall survival (OS) were analyzed using Kaplan-Meier and multivariate Cox regression models.

**Results:**

Maximal resection was achieved in 54% of patients. MGMT-methylated tumors were associated with longer OS than non-methylated cases (18.7 vs. 17.2 months; p = 0.012), while PFS2 did not differ significantly by MGMT status (4.1 vs. 3.4 months; p = 0.11). Maximal resection significantly improved PFS2 (6.27 vs. 3.02 months; p = 0.0064) and showed a trend toward better OS (p = 0.055). MGMT-methylated patients with maximal resection had the best outcomes (PFS2: 6.27 months; OS: 14.1 months). In multivariate analysis, submaximal resection predicted worse PFS2 (p < 0.001) and RS (p = 0.024), while ECOG ≥2 was associated with shorter PFS2 (p = 0.02). In non-methylated patients, resection extent and ECOG were not significant prognostic factors.

**Conclusion:**

MGMT methylation correlates with improved survival. In methylated rGB, submaximal resection and poor performance status predict worse outcomes, supporting maximal resection as a key prognostic factor.

## Highlights

• Maximal re-resection significantly prolongs PFS after recurrent glioblastoma.
• Survival benefit of complete re-resection is strongest in MGMT-methylated tumors.
• Submaximal re-resection predicts worse PFS and post-recurrence survival.
• MGMT methylation remains a key prognostic factor in recurrent glioblastoma.
• ECOG performance status independently influences progression after recurrence.

## 1 Introduction

Glioblastoma (GBM) is the most common and aggressive malignant primary brain tumor in adults, accounting for 47.7% of all central nervous system (CNS) malignant tumors in this population (Tamimi and Juweid, 2017; Kanderi et al., 2024). The annual age-adjusted incidence rate of GBM ranges from 3 to 5 per 100,000 persons, with a median age at diagnosis of 64 years (Chakrabarti et al., 2005; Ostrom et al., 2014).

The current standard treatment for newly diagnosed GBM consists of maximal safe surgical resection followed by radiotherapy in combination with concomitant and adjuvant temozolomide, an oral alkylating chemotherapeutic agent (Stupp et al., 2005). In patients with MGMT promoter–methylated tumors, an alternative treatment strategy according to the CeTeG protocol has been shown to provide benefit (Herrlinger et al., 2019; Tzaridis et al., 2021). In addition, tumor treating fields (TTF) therapy has been associated with improved survival in newly diagnosed GBM(Stupp et al., 2017; Ballo et al., 2023).

Despite these multimodal treatment approaches, tumor recurrence occurs in the majority of patients, and overall survival (OS) remains poor, with a median of only 14.6 months after initial diagnosis (Stupp et al., 2005).

At recurrence, treatment strategies are more individualized. Surgical re-resection is an option, with approximately 20–30% of patients being eligible for a second resection (Weller et al., 2017). Several studies have demonstrated the beneficial effects of re-resection (Ringel et al., 2016; Oppenlander et al., 2014), with survival outcomes largely depending on the extent of resection: complete resection of contrast-enhancing tumor is associated with improved survival compared to incomplete resection.

The alkylating agent lomustine is established as a second-line chemotherapy for recurrent GBM and has shown a survival benefit (Wick et al., 2017). Re-irradiation is another option but is not consistently offered across treatment centers (Kazmi et al., 2019). Consequently, lomustine remains the primary systemic therapy in the recurrent setting. However, the efficacy of alkylating chemotherapy is strongly influenced by the tumor's MGMT promoter methylation status, with MGMT non-methylated tumors showing significantly reduced responsiveness (Wick et al., 2017).

This raises the question of whether complete surgical resection of recurrent tumor tissue is of greater importance in MGMT non-methylated GBM, given the limited efficacy of second-line chemotherapy in this patient subgroup (Hegi et al., 2005).

The present study therefore assessed the impact of complete versus incomplete surgical resection of contrast-enhancing tumor in recurrent GBM, stratified by MGMT promoter methylation status.

## 2 Materials and methods

### 2.1 Study design

This retrospective observational study analyzed medical records of consecutive patients who underwent surgical resection for recurrent glioblastoma between January 2016 and December 2021 at a single institution. All patient data were de-identified before analysis. Data are reported in accordance with the STROBE guidelines for observational studies.

### 2.2 Patients and parameters

**Inclusion criteria** were:

1. Primary IDH-wildtype glioblastoma,
2. Histopathologically confirmed recurrent glioblastoma,
3. Surgical resection performed for recurrent glioblastoma.

**Exclusion criteria** were:

1. Initial diagnosis based on biopsy only (no resection),
2. Non-surgical management of the first recurrence,
3. Incomplete medical documentation,
4. Loss to follow-up with no available progression-free survival or overall survival data.

Demographic and clinical parameters included sex, age at recurrence surgery, and preoperative clinical symptoms. Functional status was assessed before and after each surgery using the Eastern Cooperative Oncology Group (ECOG) Performance Status Scale (Oken et al., 1982). Tumor location was categorized as frontal, temporal, parietal, occipital, deep, or cerebellar, with “deep lesions” defined as tumors located in the thalamus, basal ganglia, brainstem, or insular cortex.

Magnetic resonance imaging (MRI) features were recorded, including bi-hemispheric involvement, corpus callosum infiltration, and midline shift. Tumor focality was classified as:

• **Unifocal**: single lesion,
• **Multifocal**: connected lesions on fluid-attenuated inversion recovery (FLAIR) MRI or microscopic invasion along white matter tracts,
• **Multicentric**: ≥2 separate, non-contiguous foci (Friso et al., 2021; Picart et al., 2018; Krenzlin et al., 2024).

Adjuvant treatments were extracted from patient records.

### 2.3 Imaging and resection assessment

A postoperative MRI performed within 72 h was used to determine extent of resection (EOR). EOR was evaluated according to the RANO categories for glioblastoma (Karschnia et al., 2023a):

• **Maximal resection**: complete CE resection (0 cm^3^ CE + >5 cm^3^ nCE) or near-total CE resection (≤1 cm^3^ CE),
• **Submaximal resection**: subtotal CE resection (≤5 cm^3^ CE) or partial CE resection (>5 cm^3^ CE).

Tumor volumes (cm^3^) were manually segmented using Brainlab Elements® software (version 3.1.0, Brainlab AG, Munich, Germany) on axial, coronal, and sagittal contrast-enhanced T1-weighted images.

### 2.4 Histopathology and molecular analysis

Tumor diagnosis was established according to the WHO 2021 classification (Louis et al., 2021). MGMT promoter methylation status was determined from formalin-fixed, paraffin-embedded (FFPE) tumor samples. DNA was extracted using commercial kits, followed by bisulfite conversion. Methylation-specific PCR (MSP) was performed using primers specific for methylated and unmethylated MGMT promoter regions. For a subset of samples, pyrosequencing was performed to quantify methylation levels at individual CpG sites.

### 2.5 Survival definitions

• **Progression-free survival 1 (PFS1):** time from initial surgery to radiological progression,
• **Progression-free survival 2 (PFS2):** time from re-resection to subsequent radiological progression,
• **Overall survival (OS):** time from initial surgery to death,
• **Reoperation survival (RS):** time from re-resection to death.

Patients without documented progression or death were considered lost to follow-up.

### 2.6 Statistical analysis

Statistical analyses were performed using R software (version 4.0.4). Kaplan–Meier curves were generated for OS, RS, and PFS2. Categorical variables were compared using Chi-squared tests, while continuous variables were analyzed with ANOVA. Significant predictors were further evaluated with Tukey's HSD post-hoc tests. Stepwise multivariate Cox regression models were used to identify the optimal set of covariates. Separate Cox regression analyses were performed for MGMT-methylated and unmethylated subgroups, with PFS2 as the outcome variable.

## 3 Results

### 3.1 Patient characteristics

Patient characteristics are summarized in Table 1. A total of 95 patients who underwent surgical resection for recurrent glioblastoma were identified and analyzed. Among them, 41 tumors were MGMT promoter–methylated and 54 were non-methylated.

**Table 1:** Baseline characteristics

	MGMT Methylated	MGMT Unmethylated	Overall	p-value
	N = 41	N = 54	N = 95	
**Gender** (Female)	22 (54%)	21 (39%)	43 (45%)	0.22
**Age**	65 (IQR: 17)	59 (IQR: 18.4)	62 (IQR: 16.5)	0.067
**ECOG at Admission**				0.46
0	8 (20%)	10 (19%)	18 (19%)	
1	21 (51%)	35 (65%)	56 (59%)	
2	11 (27%)	8 (15%)	19 (20%)	
3	1 (2%)	1 (2%)	2 (2%)	
**Symptoms**				
Headache	5 (12%)	13 (24%)	18 (19%)	0.23
Seizures	6 (15%)	12 (22%)	18 (19%)	0.5
Hemiparesis	10 (24%)	10 (19%)	20 (21%)	0.66
Aphasia	16 (39%)	12 (22%)	28 (29%)	0.12
Nausea	0 (0%)	3 (6%)	3 (3%)	0.26
Hemianopsia	2 (5%)	6 (11%)	8 (8%)	0.46
**MRI features**				
Bihemispheric	1 (2%)	2 (4%)	3 (3%)	1
Corpus Callosum Invasion	3 (7%)	10 (19%)	13 (14%)	0.14
Midline Shift	15 (37%)	15 (28%)	30 (32%)	0.49
**Lesion Focality**				
Unifocal	36 (88%)	49 (91%)	85 (89%)	0.9
Multifocal	3 (7%)	0 (0%)	3 (3%)	0.077
Multicentric	2 (5%)	4 (7%)	6 (6%)	0.7
**Extent of Re-Resection**				0.55
Maximal Resection	22 (53.7%)	30 (55.6%)	52 (54.7%)	
Submaximal Resection	19 (46.3%)	24 (44.4%)	43 (45.3%)	
**Adjuvant Therapy after re-resection**				0.0021
Radiotherapy	2 (4.8%)	2 (3.7%)	4 (4.2%)	
Chemotherapy	30 (73.2%)	42 (77.8%)	72 (75.8%)	
Radiochemotherapy	1 (2.5%)	4 (7.4%)	5 (5.3%)	
No therapy	8 (19.5%)	6 (11.1%)	14 (14.7%)	

**Fig. 1:**
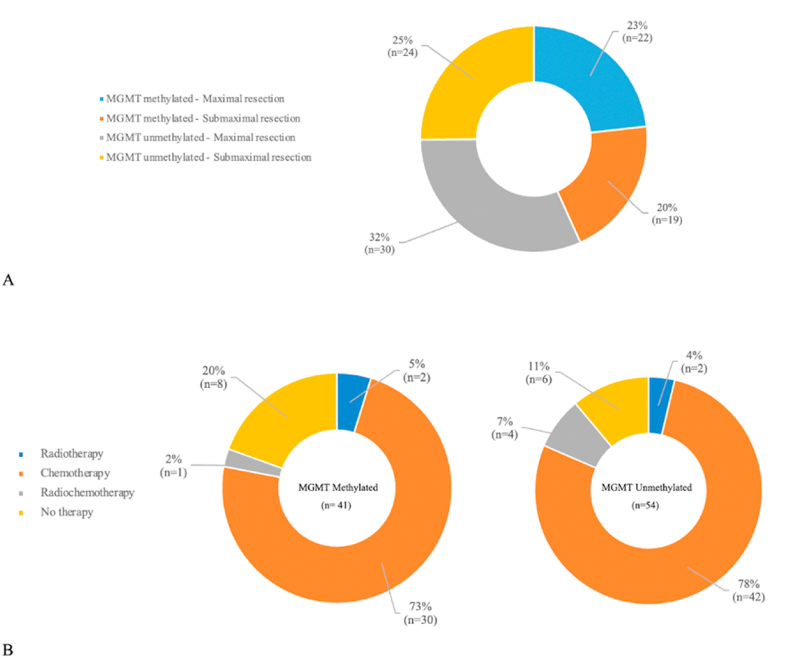
Distribution of patients by MGMT promoter methylation status and extent of resection (A) and adjuvant therapy (B).

Prior to re-resection, 64 patients (78%) had an ECOG Performance Status of 0–1, 19 patients (20%) had ECOG 2, and 2 patients (2%) had ECOG 3. Maximal re-resection was achieved in 52 patients (54.7%), including 22 (53.7%) with methylated tumors and 30 (55.6%) with non-methylated tumors. Submaximal re-resection was achieved in 43 patients (45.3%), including 19 (46.3%) with methylated tumors and 24 (44.4%) with non-methylated tumors (Table 1). The distribution of patients stratified by MGMT status and extent of re-resection is shown in Fig. 1.

### 3.2 Prognostic impact of MGMT promotor methylation and survival outcomes

**Fig. 2:**
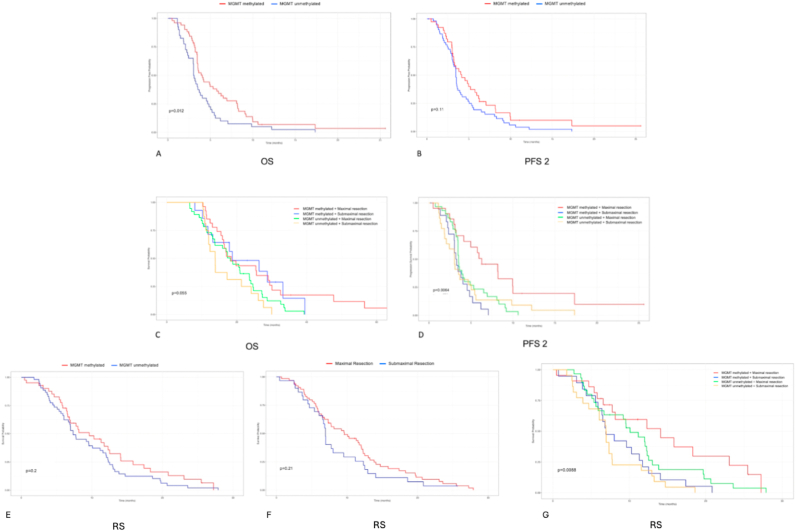
Kaplan–Meier survival analyses for glioblastoma patients stratified by MGMT promoter methylation status and extent of resection. Panels A–B show overall survival (OS) and progression-free survival 2 (PFS2) stratified by MGMT methylation status. Panels C–D show OS and PFS2 further stratified by combined MGMT status and extent of resection. Panels E–G present reoperation survival (RS) according to MGMT status (E), extent of resection (F), and their combined stratification (G).

Kaplan–Meier analysis demonstrated a significant association between MGMT status and overall survival (OS). Patients with MGMT-methylated GBMs had longer OS compared to those with non-methylated GBMs (18.7 vs. 17.2 months, *p* = 0.012;Table 2,Fig. 2A). Progression-free survival 2 (PFS2) did not differ significantly between the groups (4.1 vs. 3.4 months, *p* = 0.11;Fig. 2B).

**Table 2:** Survival outcomes by MGMT methylation status and extent of resection.

	Progression free survival 1	Progression free survival 2	Overall survival	Reoperation Survival
Overall, median survival (months)	6.9 (95%CI 6.1 - 8.4)	3.5 (95%CI 3.3 - 4.2)	17.3 (95%CI 15.9 - 21.3)	7.8 (95%CI 6.9 - 11.3)
Methylated overall, median survival (months)	8.4 (95%CI 6.3 - 11.8)	4.1 (95%CI 3.2 - 6.2)	18.7 (95%CI 16.4 - 29)	9.6 (95%CI 6.8 - 14.1)
Methylated complete, median survival (months)	8.4 (95%CI 7.0 - 12.7)	6.3 (95%CI 4.1 - NA)	18.4 (95%CI 16.3 - 30.1)	14.1 (95%CI 7.8 - NA)
Methylated incomplete, median survival (months)	8.5 (95%CI 5.5 - 25.1)	3.2 (95%CI 3 - 4.8)	18.7 (95%CI 13.1 - NA)	6.9 (95%CI 6.4 - 12.5)
Difference methylated complete vs incomplete	HR: 1.0 (95%CI 0.5 - 1.9)	HR: 3.6 (95%CI 1.7 - 8)	HR: 1.1 (95%CI 0.5 - 2.2)	HR: 2.5 (95%CI 1.2 - 5.1)
Unmethylated overall, median survival (months)	6.1 (95%CI 4.9 - 7.4)	3.4 (95%CI 3.2 - 4)	17.2 (95%CI 13.7 - 21.3)	7.3 (95%CI 6.7 - 11.6)
Unmethylated complete, median survival (months)	6.1 (95%CI 4.7 - 9)	3.5 (95%CI 3.4 - 4.9)	18.1 (95%CI 13.7 - 24)	10.1 (95%CI 6.7 - 12.5)
Unmethylated incomplete, median survival (months)	5.6 (95%CI 4.8 - 10.4)	3 (95%CI 2.1 - 5)	13.8 (95%CI 11.9 - 26.1)	6.9 (95%CI 6 - 7.8)
Difference unmethylated complete vs incomplete	HR: 1.3 (95%CI 0.7 - 2.4)	HR: 1.7 (95%CI 1.1 - 2.6)	HR: 1.4 (95%CI 0.8 - 2.5)	HR: 2.0 (95%CI 1.3 - 3.1)

^1^HR = Hazard Ratio, CI = Confidence Interval.

When stratified by MGMT status and extent of re-resection, a trend toward significance was observed (*p* = 0.055;Fig. 2C). Median OS among MGMT-methylated patients was 18.4 months with maximal re-resection and 18.7 months with submaximal re-resection. In contrast, MGMT-non-methylated patients had a median OS of 18.1 months with maximal re-resection versus 13.8 months with submaximal re-resection.

For PFS2, MGMT-methylated patients who underwent maximal re-resection demonstrated the longest median survival (6.27 months) compared with those receiving submaximal re-resection (3.24 months). In MGMT non-methylated patients, PFS2 was shorter, with 3.52 months for maximal re-resection and 3.02 months for submaximal re-resection. The differences across groups were statistically significant (*p* = 0.0064;Fig. 2D).

Reoperation survival (RS) analysis showed a trend toward improved outcomes in MGMT-methylated patients (median 9.6 months) compared with non-methylated patients (median 7.3 months), though this did not reach statistical significance (*p* = 0.20;Fig. 2E). Patients undergoing maximal re-resection had a median RS of 9.6 months versus 6.9 months for submaximal re-resection, also not statistically significant (*p* = 0.21;Fig. 2F).

When stratified into four subgroups based on MGMT status and re-resection extent, significant differences in RS were observed (*p* = 0.0088;Fig. 2G). The longest RS was observed in MGMT-methylated patients with maximal re-resection (14.1 months), followed by MGMT non-methylated patients with maximal re-resection (10.1 months). Shorter RS was seen in the submaximal resection groups: 6.9 months (methylated) and 6.7 months (non-methylated).

Comparing the survival benefit of maximal over submaximal re-resection revealed a greater effect in MGMT-methylated tumors (7.2 months) than in non-methylated tumors (3.4 months), indicating that complete resection confers stronger benefit in the methylated subgroup.

### 3.3 Cox and multivariate analyses

In the multivariate Cox regression analysis of PFS2 for MGMT-methylated tumors, higher ECOG score at recurrence was associated with increased risk of progression (HR = 2.82, 95% CI: 1.15–6.89, *p* = 0.02). Submaximal re-resection was also independently associated with worse PFS2 (HR = 6.44, 95% CI: 2.41–17.20, *p* < 0.001).

For RS, in patients with MGMT-methylated glioblastoma, submaximal resection at first relapse was the only independent adverse prognostic factor (Table 3). Compared with maximal resection, submaximal resection was associated with a shorter RS (HR = 2.60; 95% CI 1.13–5.96; p = 0.024). Age (HR = 1.02; 95% CI 0.98–1.05; p = 0.3), ECOG status at relapse (HR = 0.85; 95% CI 0.38–1.90; p = 0.7), and the use of radiotherapy (HR = 1.79; 95% CI 0.48–6.64; p = 0.4) or chemotherapy (HR = 0.97; 95% CI 0.38–2.43; p > 0.9) at relapse were not significantly associated with RS.

**Table 3:** Multivariable Cox regression analysis of reoperation survival (RS) stratified by MGMT promoter methylation status.

Variable	MGMT Methylated		MGMT Unmethylated	
	HR (95%Cl)^a^	p-value	HR (95%Cl)^a^	p-value
**Age**	1.02 (0.98-1.05)	0.3	1.04 (1.01-1.07)	0.017
**ECOG at relapse**	0.85 (0.38-1.90)	0.7	1.28 (0.81-2.07)	0.3
**EOR at first relapse**				
Submaximal Resection	2.60 (1.13-5.96)	0.024	1.64 (0.90-2.98)	0.11
**Radiotherapie at relapse**	1.79 (0.48-6.64)	0.4	-	-
**Chemotherapy at relapse**	0.97 (0.38-2.43)	>0.9	0.67 (0.27-1.67)	0.4

a HR = Hazard Ratio, CI = Confidence Interval.

Among patients with MGMT-unmethylated glioblastoma, age emerged as the only independent predictor (HR = 1.04; 95% CI 1.01–1.07; p = 0.017). ECOG status at relapse (HR = 1.29; 95% CI 0.81–2.07; p = 0.3), submaximal resection (HR = 1.64; 95% CI 0.90–2.98; p = 0.11), and chemotherapy at relapse (HR = 0.67; 95% CI 0.27–1.68; p = 0.4) did not reach statistical significance.

## 4 Discussion

The key finding of this study is that the survival benefit associated with maximal re-resection in recurrent glioblastoma is not uniform, but differs according to MGMT promoter methylation status. The extent of surgical resection at recurrence emerged as a key determinant of outcome, particularly in MGMT-methylated tumors, where maximal re-resection significantly prolonged PFS after recurrence and was strongly associated with improved post-recurrence survival in multivariate analysis. Although maximal resection also conferred some benefit in MGMT-unmethylated patients, the effect was smaller and did not reach statistical significance.

Taken together, these findings highlight the prognostic relevance of both MGMT status and extent of re-resection, suggesting that complete re-resection is especially critical in patients with MGMT-methylated recurrent glioblastoma, but less impactful in those with unmethylated MGMT promoters.

Importantly, our findings challenge the intuitive assumption that maximal resection would be most critical in MGMT-unmethylated tumors, where systemic treatment options are limited. Instead, we observed a more pronounced survival benefit in MGMT-methylated patients. This suggests that the biological context of the tumor may influence the effectiveness of surgical cytoreduction, potentially through interactions with adjuvant therapies or inherent tumor behavior.

The management of recurrent GBM remains one of the most challenging aspects of treatment, leading to a critical need for prognostic biomarkers that can guide individualized treatment. One such biomarker, O6-methylguanine-DNA methyltransferase (MGMT) promoter methylation has its prognostic and predictive significance in newly diagnosed glioblastoma. MGMT is a DNA repair enzyme that counteracts the effects of alkylating agents like TMZ, the standard chemotherapy for GBM. Methylation of the MGMT promoter silences the gene, thereby enhancing the cytotoxic effects of TMZ by impairing the tumor cell's ability to repair DNA damage (Hegi et al., 2005).

In recurrent glioblastoma, where treatment options are often limited to the alkylating chemotherapeutic agent lomustine, the status of MGMT methylation could play an even more critical role due to the lack of a Standard irradiation (Choi et al., 2021). Response to lomustine treatment does depend on MGMT methylation status and in non-methylated tumors lomustine has potentially no relevant effect. Our study shows that MGMT promoter methylation is associated with significantly improved overall survival in recurrent glioblastoma, with a median OS of 18.7 months in the methylated group compared to 17.2 months in the unmethylated group (p = 0.012). This supports the continued relevance of MGMT methylation as a prognostic marker in the recurrent GBM, as reported in recent studies that MGMT methylation improves the sensitivity of GBM to TMZ, even after recurrence, which is reflected in better overall survival (Weller et al., 2020, 2021; Karschnia et al., 2023b).

However, MGMT methylation did not significantly influence PFS2 (4.1 vs. 3.4 months, p = 0.11). This suggest while it continues to influence OS, its effect on tumor progression after recurrence is more limited. Previous studies have emphasized the multifactorial nature of tumor progression and suggest that tumor biology during progression may play a greater role in determining PFS(Weller et al., 2020; Han et al., 2014; Sun and Kim, 2022). Furthermore, noted that while MGMT methylation improves response to chemotherapy, its influence on tumor progression may decrease in the context of tumor heterogeneity and resistance mechanism that emerge after recurrence (Ashkan et al., 2023). Our results contrast with clear impact of MGMT methylation on PFS in newly diagnosed glioblastoma (Stupp et al., 2005) and suggest that the role of MGMT methylation in the recurrent GBM may be more complex.

ECOG performance status remains a robust predictor of survival in GBM. In our study, patients with higher ECOG scores (≥2) at recurrence hat a significantly increased risk of progression (HR = 2.82, p = 0.02). Patients with better functional status are more likely to tolerate aggressive treatments, including re-resection and chemotherapy, leading to improved survival (Navarria et al., 2019).

Due to the limited response of MGMT non-methylated tumors upon treatment with lomustine the question arises whether the extent of the surgical resection has a stronger impact in non-methylated tumors non-responsive to alkylating chemotherapy. Our study showed that the extent of re-resection is an extremely important prognostic factor. However, the treatment effect of a complete resection was not more pronounced in the non-methylated group, moreover, patients with methylated tumors showed a more pronounced benefit from a maximal re-resection. Patients who underwent submaximal re-resection had a significantly worse progression free survival after recurrence (HR = 6.44, p < 0.001) and reoperation survival in the MGMT-methylated group (p = 0.024).

These findings suggest that the benefit of maximal resection should be interpreted in the context of tumor biology, particularly MGMT status. The ability to achieve maximal tumor resection may enhance the efficacy of subsequent therapies by reducing the tumor burden and allowing for a more robust therapeutic response.

Chemotherapy represented an important component of treatment in the majority of our cohort (76% of patients underwent chemotherapy after re-resection). However, in our multivariate analysis, chemotherapy did not demonstrate a significant association with overall survival in either MGMT-methylated or non-methylated patients (p = 0.93 and p = 0.66, respectively). These findings contrast with established role of MGMT methylation in predicting responsiveness to alkylating chemotherapy in newly diagnosed and recurrent glioblastoma. A possible explanation lies in the complex tumor biology at recurrence, including acquired resistance mechanisms, intratumoral heterogeneity, and treatment-related molecular evolution (Taylor and Schiff, 2015).Nevertheless, prior studies have reported a survival benefit from continued alkylating chemotherapy in selected patients with recurrent GBM and MGMT promoter methylation (Rivera et al., 2010) Our results therefore suggest that survival impact of the chemotherapy in the recurrent setting is less pronounced and may be modulated by other clinical and molecular factors.

The limitations of our study include its retrospective nature and relatively small sample size, which may reduce the statistical power and generalizability of our findings. Additionally, variability in treatment regimens, including heterogeneity of adjuvant therapies could have impact on the results. Further studies should focus on larger, prospective cohort to further validate the role of MGMT methylation in recurrent GBM.

## 5 Conclusion

Our findings suggest that the survival benefit of maximal re-resection in recurrent glioblastoma is modulated by MGMT promoter methylation status. Rather than representing a uniform principle, the impact of surgical extent appears to differ across molecular subgroups, supporting a more individualized, biologically informed surgical decision-making approach.

## Ethics approval

Data acquisition and analysis were performed anonymously and were approved by the Ethics Committee of the Medical Association of Rhineland Palatinate, Germany. According to local laws, further consent is not necessary for retrospective analysis.

## Author contributions

Dragan Jankovic: Conceptualization; Data curation; Formal analysis; Methodology; Writing—original draft. Santhosh G. Thavarajasingam: Data curation; Methodology. Ahmed Salih: Data curation; Formal analysis. Daniel Scurtu: Data curation. Alice Dauth: Data curation; Harald Krenzlin, Darius Kalasauskas: Methodology. Writing—review & editing. Andreas Kramer: Writing—review & editing. Marc A. Brockmann: Writing—review & editing. Clemens Sommer, Petra Leukel: Writing—review & editing. Marcus Stockinger: Writing—review & editing. Naureen Keric: Conceptualization, Writing—review & editing. Florian Ringel: Conceptualization; Supervision; Writing—review & editing. All authors reviewed the manuscript.

## Ethics approval

Since this study involved a retrospective analysis of routinely collected and fully anonymized data, no informed consent or ethical committee approval was required, in accordance with §§36 and 37 of the Landeskrankenhausgesetz Rheinland-Pfalz.

## Funding

The authors declare that no funds, grants, or other support were received during the preparation of this manuscript.

## Declaration of competing interest

The authors declare that they have no known competing financial interests or personal relationships that could have appeared to influence the work reported in this paper.

## Data Availability

The datasets generated during and/or analyzed during the current study are available from the corresponding author on reasonable request.
